# An autophagy-associated lncRNAs model for predicting the survival in non-small cell lung cancer patients

**DOI:** 10.3389/fgene.2022.919857

**Published:** 2022-09-02

**Authors:** Jing Hu, Pei-Jin Zhang, Di Zhang, Zhao-Hui Chen, Xu-Chen Cao, Yue Yu, Jie Ge

**Affiliations:** ^1^ The First Department of Breast Cancer, Tianjin Medical University Cancer Institute and Hospital, National Clinical Research Center for Cancer, Tianjin, China; ^2^ Key Laboratory of Cancer Prevention and Therapy, Tianjin, China; ^3^ Tianjin’s Clinical Research Center for Cancer, Tianjin, China; ^4^ Key Laboratory of Breast Cancer Prevention and Therapy, Tianjin Medical University, Ministry of Education, Tianjin, China; ^5^ Beijing Tsinghua Changgung Hospital, School of Clinical Medicine, Tsinghua University, Beijing, China

**Keywords:** autophagy, lncRNAs, NSCLC, TCGA, prognostic signature

## Abstract

Long non-coding RNAs (lncRNAs) can influence the proliferation, autophagy, and apoptosis of non-small cell lung cancer (NSCLC). LncRNAs also emerge as valuable prognostic factors for NSCLC patients. Consequently, we set out to discover more autophagy-associated lncRNAs. We acquired autophagy-associated genes and information on lncRNAs from The Cancer Genome Atlas database (TCGA), and the Human Autophagy Database (HADb). Then, the prognostic prediction signature was constructed through using co-expression and Cox regression analysis. The signature was constructed including 7 autophagy-associated lncRNAs (ABALON, NKILA, LINC00941, AL161431.1, AL691432.2, AC020765.2, MMP2-AS1). After that, we used univariate and multivariate Cox regression analysis to calculate the risk score. The survival analysis and ROC curve analysis confirmed good performances of the signature. GSEA indicated that the high-risk group was principally enriched in the adherens junction pathway. In addition, biological experiments showed that ABALON promoted the proliferation, metastasis and autophagy levels of NSCLC cells. These findings demonstrate that the risk signature consisting of 7 autophagy-associated lncRNAs accurately predicts the prognosis of NSCLC patients and should be investigated for potential therapeutic targets in clinic.

## Introduction

Lung cancer is the most common malignant disease. Among these cases, 84% are classified as NSCLC with the remaining 16% classified as small cell lung cancer (SCLC) (Torre et al., 2012). Because of the advent of new targeted drugs and advances in therapy, clinical treatment of lung cancer has made great progress. However, the attendant problems also increased, such as inherent resistance to both chemotherapy and radiation therapy (Hirsch et al., 2017); (Yang et al., 2019), and it is becoming increasingly challenging to assess the prognosis of NSCLC patients. Therefore, it is imperative to confirm biomarkers of prognosis in NSCLC.

As an intracellular catabolic degradation process, autophagy contributes to normal cell physiology by eliminating damaged proteins and other cell components (Li et al., 2020). There is a great deal of studies showing that autophagy also participates in various pathological processes, for example liver disorders and infectious diseases (Ueno and Komatsu, 2017); (Levine and Kroemer, 2019); (Choi et al., 2018). Moreover, a growing body of research suggests autophagy plays a dual role in cancer. Autophagy can suppress chronic tissue damage to inhibit tumorigenesis. It could promote longevity of normal cells *via* regulating the quality of proteins and organelles, promoting the stability of the genome, or a combination of these factors (Barnard et al., 2016); (Mizushima and Levine, 2020). However, autophagy can also maintain the function of mitochondria and reduce DNA damage to enhance the ability to resist stress and apoptosis in cancer cells (White et al., 2015); (Bravo-San Pedro et al., 2017). Many studies have also shown that modulators of autophagy can prevent NSCLC from developing (Bai et al., 2019). However, other research shows that upregulation of autophagy can promote tumorigenesis and immune escape of cancer cells (Ma et al., 2020). Ma et al. (2013) SKIL promoted tumorigenesis and immune escape of NSCLC cells through upregulation of TAZ/autophagy axis and inhibition on downstream STING pathway. Therefore, it is important to find autophagy-associated transcripts which are considered as valuable biomarkers for diagnosis and prognosis in NSCLC.

LncRNAs, more than 200 nucleotides, is a class of RNA transcripts and does not code proteins. LncRNAs participate in some fundamental cancer-related processes through transcriptional for or post-transcriptional regulation, such as proliferation, migration, survival, and metastasis (Fang and Fullwood, 2016); (Slack and Chinnaiyan, 2019). Furthermore, a great number of studies identified that lncRNAs regulated autophagy (Xu et al., 2019); (Wu et al., 2021); (Kopp and Mendell, 2018). For example, Wang et al. (2019a) proved the lncRNA LINRIS could block the degradation of IGF2BP2 and suppress the proliferation of CRC *via* the ubiquitination-autophagy pathway. The lncRNA NBAT1 inhibits autophagy by inhibiting ATG7 in NSCLC (Zheng et al., 2018). Hence, it is meaningful to confirm major lncRNAs connected with autophagy and prognosis of NSCLC.

In our study, we systematically analyzed lncRNAs data of NSCLC patients in TCGA. We also founded an accurate prognostic signature of 7 autophagy-associated lncRNAs and assessed their ability to precisely predict the prognosis. It was verified that the downregulation of ABALON affected proliferation, metastasis, and autophagy of NSCLC cells by experimental validation. We provide a novel prognostic signature consisting of 7 autophagy-associated lncRNAs, which may also be potential therapeutic targets.

## Materials and methods

### Patient data sets

The transcriptome profiles and corresponding clinical date of 1,145 NSCLC patients (1,037 cases NSCLC patients and 108 healthy controls) were extracted from TCGA (https://portal.gdc.cancer.gov/), and all genes ID were transformed. LncRNAs and protein-coding genes were annotated and classified *via* the Ensembl human genome browser. We acquired data of autophagy-associated genes from the HADb (https://www.autophagy.lu/). We extract potential autophagy-associated lncRNAs *via* pearson correlation analysis, |*R* ^2^| > 0.3 and *p*-value < 0.001 were defined as thresholds.

### Construction and evaluation of an autophagy-associated long non-coding RNAs prognostic signature

We combined the expression level of autophagy-associated lncRNAs with the corresponding survival results in TCGA. Autophagy-associated lncRNAs were confirmed by univariate Cox regression analysis. The calculation formula of risk score for every patient was: risk score = $\overset{n}{\underset{k=1}{\sum }}coef\left(k\right)*lncRNA\left(k\right)$ , where coef (k) and lncRNA (k) respectively represent the regression coefficient and expression level of corresponding autophagy-associated lncRNA. These lncRNAs data were divided into two groups based on the median risk score. Then we constructed the best prognostic risk model of autophagy-associated lncRNAs *via* multivariate Cox regression analysis. Subsequently, we evaluated the survival difference in these two groups *via* Kaplan-Meier (KM) survival analysis. We explored the correlation between clinical factors and risk score. *R* packages “survival” and “forestplot” were performed to visualize the forest plot respectively. Finally, we draw ROC curves to estimate the predictive value of different clinical pathological factors.

### Establishment of the long non-coding RNA-mRNA co-expression network

The relationship between autophagy-associated lncRNAs and their corresponding mRNAs was explored through a co-expression network and Sankey diagram. We extracted the mRNAs that were associated with autophagy-associated lncRNAs *via* Pearson correlation coefficients, the absolute threshold coefficient value >0.3. The network was visually analyzed by cytoscape software (v 3.7.1) and ggalluvial *R* package.

### Estimation and construction of nomogram

A nomogram survival prediction model of NSCLC patients was constructed using the “survival” and “rms” packages by combining risk score and expression of autophagy-associated lncRNAs. We then constructed the ROC curves and calculated the AUC values of this nomogram.

### Functional enrichment analysis

Perform GSEA to find expression changes in predefined genomes, rather than individual genes. We verified the enrichment of differentially expressed gene sets between the low- and high-risk groups by GSEA (v4.1.0). These two groups were enriched in different signaling pathway.

### Cell culture

Beas-2B, A549, NCI-H292, NCI-H460, and NCI-H1299 cell lines were acquired from American Tissue Culture Collection (Beijing, China). Beas-2B cells were cultured in Leibovitz’s L-15 medium with 10% fetal bovine serum (FBS, Corning Incorporated) at 37°C in a 5% CO_2_ incubator. Other cell lines were cultured in RPMI 1640 with 10% FBS at 37°C in 5% CO_2_ incubator.

### RNA extraction and qRT-PCR

Total RNA was isolated from cells using the TRIzol reagent (Beyotime, Shanghai, China). The total RNA was reversed to cDNA by the PrimeScript RT reagent Kit (Takara, Japan). QRT-PCR was performed to detect the expression of ABALON using SYBR Green Mixture (Tli RNaseH Plus) (Takara, Japan) and gene specific primers. We collected data from a Roche LightCycler 480 PCR system. The results were normalized with GAPDH as an internal control. The primer sequences were as follows: GAPDH (forward: CGG​AGT​CAA​CGG​ATT​TGG​TCG​TAT; reverse: AGC​CTT​CTC​CAT​GGT​GGT​GAA​GAC), ABALON (forward: CTC​TCT​CTT​GCA​CGC​CCC​TTG; reverse: CCT​GGG​CTG​GTG​CTT​AAA​TAG​A).

### Transient transfection and small interfering RNAs

The siRNA targeting ABALON and Control were purchased from RiboBio (Guangzhou, China). When the cell confluency reaches 35%, we use FuGENE HD Transfection Reagent (Promega) to transiently transfect siRNAs (100 nm) in the cells. The medium containing 10% FBS was refreshed after 12 h.

### Proliferation and metastasis assays

EDU, MTT and colony formation assays were used to detect cell proliferation. Transwell and wound healing/scratch assays were used to estimate cell metastasis. Detailed procedures were listed in the Supplementary Material.

### Western blot analysis

Cellular protein was extracted using RIPA lysis buffer (Solarbio, China). Proteins were measured by the BCA method (Thermo scientific, United States) and separated on the SDS-PAGE gels. The protein bands were then transferred into PVDF membranes. Then the bands were blocked with 5% skim milk at room temperature for 1 h and incubated with primary antibodies overnight at 4°C. The bands were incubated with a corresponding secondary antibody for 1 h at room temperature. The protein bands were then detected using the ECL reagent (Millipore, United States). The antibodies were listed in the Supplementary Material.

### Statistical analysis

Data are presented as the means ± SD. Each experiment has a minimum of 3 replicates. Statistical analyses were executed using *R* Studio (version 4.0.3) and SPSS software (IBM Corp, United States). *p*-values < 0.05 were regarded as statistically significant.

## Results

### Identification of prognostic autophagy-associated long non-coding RNAs in non-small cell lung cancer patients

**FIGURE 1:**
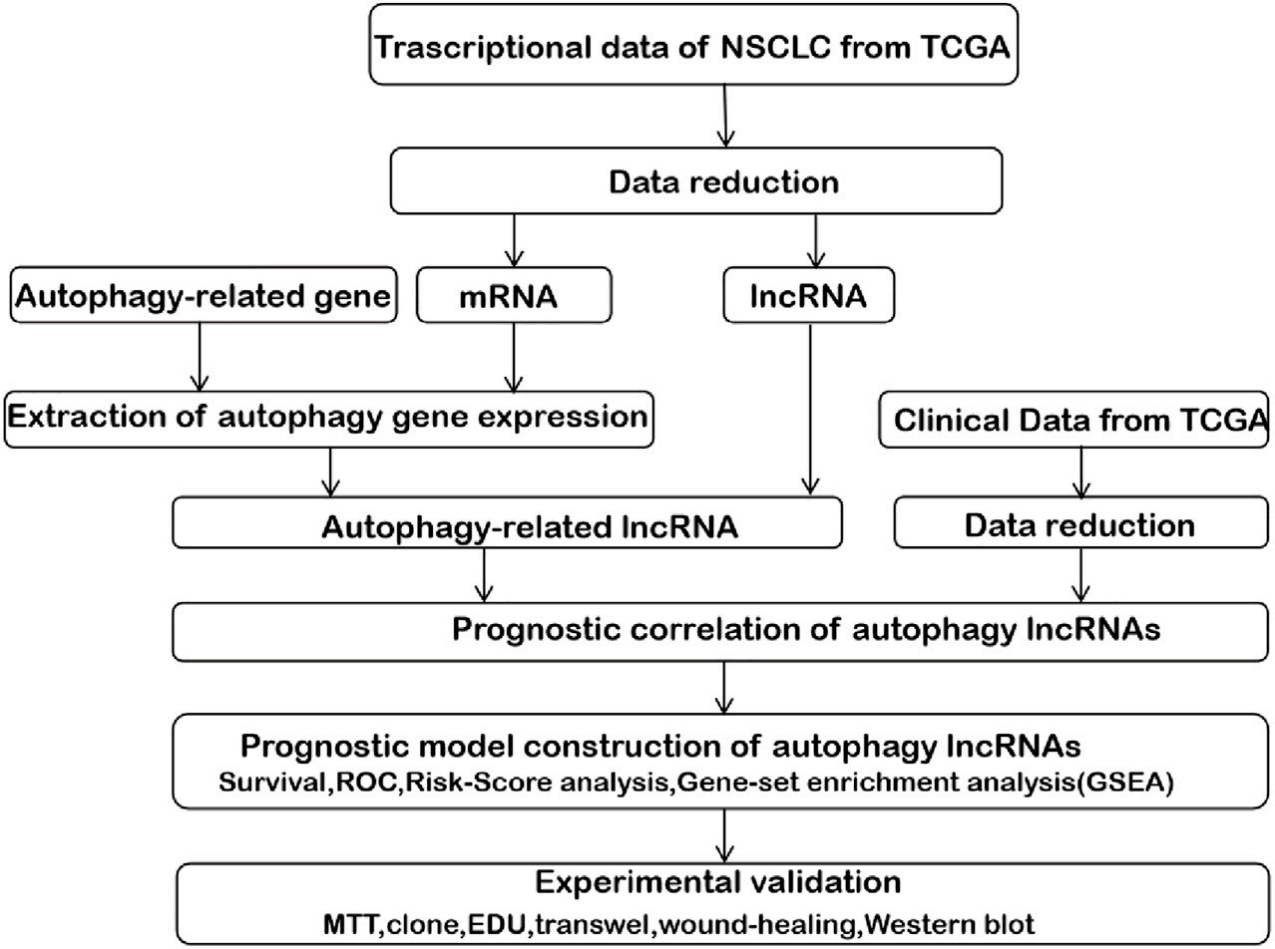
The comprehensive prognostic analysis framework of autophagy-associated lncRNAs in NSCLC based on the TCGA database.

**FIGURE 2:**
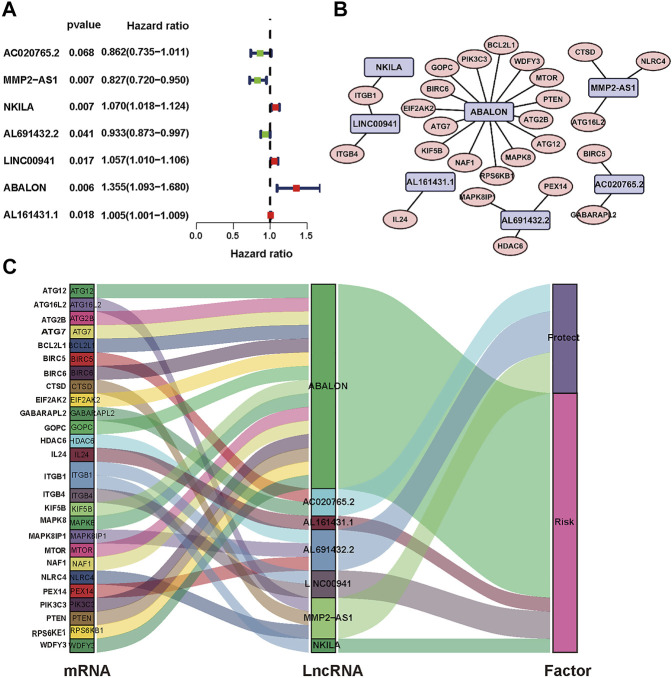
Identification of prognostic autophagy-associated lncRNAs in NSCLC patients. **(A)** Multivariate Cox analysis to establish the prognostic signature. **(B)** Establishment of the co-expression network in NSCLC. Blue represents lncRNAs and pink represents mRNAs. **(C)** The Sankey diagram shows the connection degree between 26 mRNAs and 7 autophagy-associated lncRNAs.

The analysis framework of this research is performed in Figure 1. We extracted 141432 lncRNAs data sets from TCGA and 232 autophagy-associated genes from HADb in NSCLC. Then, we identified 1,496 autophagy-associated lncRNAs *via* conducting pearson correlation analysis (*p* < 0.001). Within them, 12 autophagy-associated lncRNAs were closely correlated with the survival of NSCLC patients from TCGA *via* univariate Cox regression analysis (*p* < 0.005; Table 1). Multivariate Cox regression analysis further revealed 7 autophagy-associated lncRNAs were good candidates for constructing the diagnostic signature. Among these 7 autophagy-associated lncRNAs, AC020765.2, MMP2-AS1, and AL691432.2 were regarded as protective factors [hazard ration (HR) < 1], while the remaining 4 lncRNAs, NKILA, LINC00941, ABALON, and AL161431.1, were considered to be risk factors (HR > 1) (Figure 2A). The relationships between these autophagy-associated lncRNAs and mRNAs are displayed in Figures 2B,C.

**TABLE 1:** The prognostic effect of autophagy-related lncRNAs by univariate Cox analysis.

lncRNA	HR	HR.95L	HR.95H	*p*-value
AC020765.2	0.7915	0.6782	0.9238	0.003
AP000695.1	1.151	1.051	1.2605	0.0024
MMP2-AS1	0.8172	0.7124	0.9373	0.0039
AC068338.3	0.7071	0.5624	0.889	0.003
NKILA	1.0806	1.0339	1.1294	0.0006
AL691432.2	0.9098	0.8539	0.9693	0.0035
LINC00941	1.1098	1.0712	1.1499	0.0003
ABALON	1.4231	1.1691	1.7323	0.0004
AL161431.1	1.0057	1.002	1.0095	0.0025
AC135050.6	0.9733	0.9552	0.9917	0.0047
CRNDE	0.9616	0.9388	0.9849	0.0014
AC012615.1	0.8814	0.8098	0.9592	0.0035

### Prognosis evaluation of the autophagy-associated long non-coding RNA signature in non-small cell lung cancer

**FIGURE 3:**
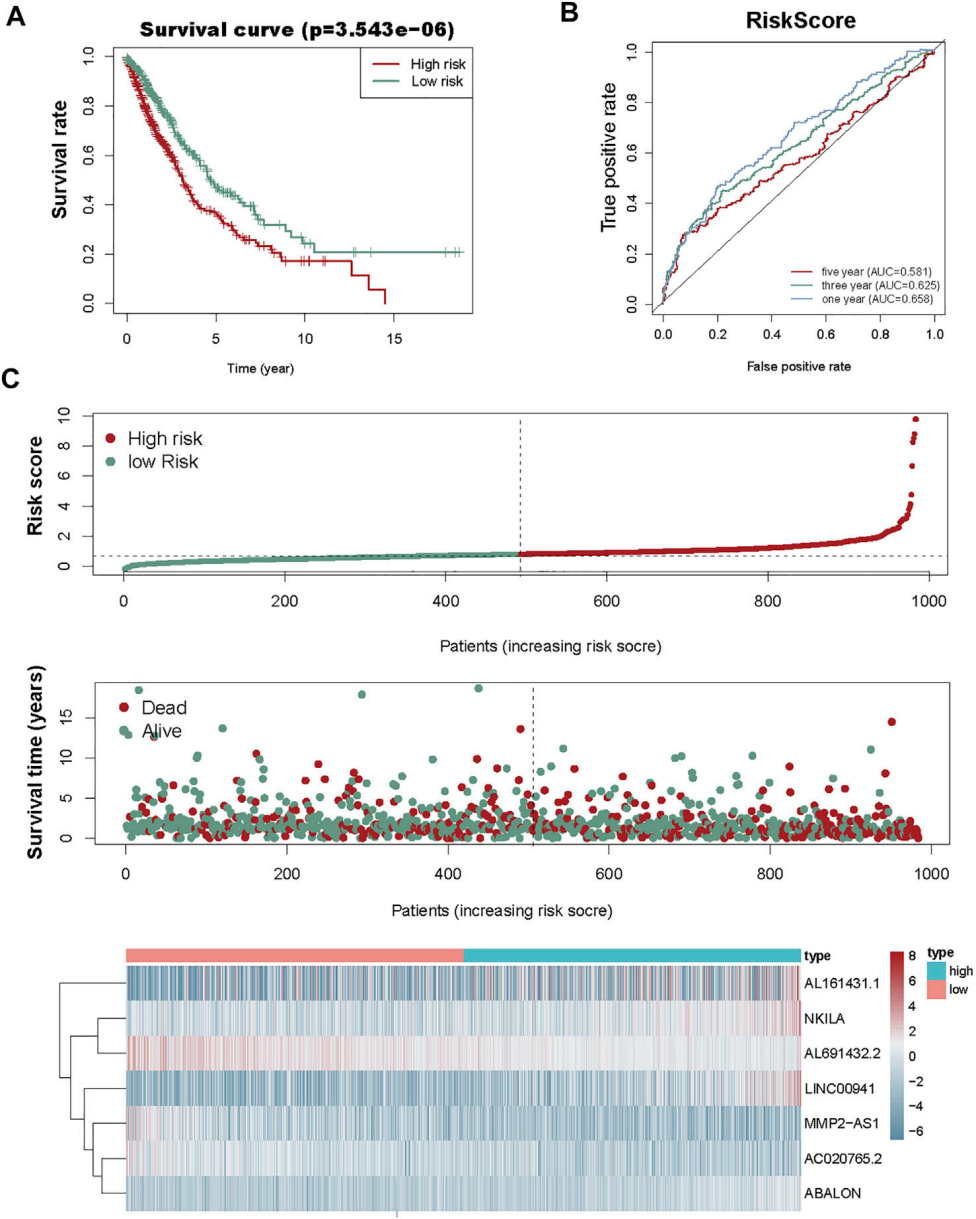
Risk score analysis of the prognostic model of these seven autophagy-associated lncRNAs. **(A)** KM survival analysis for high- and low-risk groups. Red, high risk. Green, low-risk. **(B)** ROC analysis to evaluate the predictive ability of the model. **(C)** Risk score distribution (top), survival status distribution (middle), and heat map of 7 autophagy-associated lncRNAs (bottom).

We used the prognostic risk scoring method to construct a model consisting of 7 autophagy-associated lncRNAs. The formula of the risk score was as follows: risk score= (0.0674 × NKILA) + (−0.1483 × AC020765.2) + (−0.1896 × MMP2-AS1) + (−0.0692 × AL691432.2) + (0.0555 × LINC00941) + (0.3039 × ABALON) + (0.0049 × AL161431.1) (*p* < 0.05; Table 2). Subsequently, we figured out the risk score for NSCLC patients. These samples from 1,145 NSCLC were divided into high- and low-risk groups *via* this median risk score as the cutoff. KM survival curve analysis revealed the OS of high-risk NSCLC patients was obviously shorter than of low-risk NSCLC patients (*p* < 0.001). The 3-year OS rates of the high and low-risk groups were 51.6% and 65.9%. Similarly, the 5-year OS rates of these two groups were 36.2% and 46.9% (Figure 3A), respectively. We next evaluated the prognostic power of the autophagy-associated lncRNAs model by ROC analysis. The AUC values were respectively 0.658, 0.625, and 0.581 for 1-, 3-, and 5-year OS (Figure 3B). Thus, the signature demonstrated a precise prognostic value. The risk curve of the prognostic signature and scatterplot indicated that the mortality was correlated with the risk score. The heatmap showing the expression of 7 autophagy-associated lncRNAs in NSCLC samples exhibited that NKILA, LINC00941, ABALON, and AL161431.1 were high in the high-risk group, whereas AC020765.2, MMP2-AS1, and AL691432.2 were low in the low-risk group. The risk curve, the scatterplot, and the heat map were shown in Figure 3C.

**TABLE 2:** Correlation between autophagy genes and lncRNAs in NSCLC.

Autophagy genes	lncRNA	Correlation	*p*-value
BIRC5	AC020765.2	0.35	3.32E-31
GABARAPL2	AC020765.2	0.30	4.15E-23
ATG16L2	MMP2-AS1	0.38	1.23E-37
CTSD	MMP2-AS1	0.34	6.07E-30
NLRC4	MMP2-AS1	0.37	2.17E-34
ITGB1	NKILA	0.30	4.58E-23
HDAC6	AL691432.2	0.31	8.37E-24
MAPK8IP1	AL691432.2	0.38	3.69E-36
PEX14	AL691432.2	0.39	4.15E-39
ITGB1	LINC00941	0.32	1.16E-26
ITGB4	LINC00941	0.31	1.03E-24
ATG12	ABALON	0.32	3.30E-26
ATG2B	ABALON	0.39	8.16E-40
ATG7	ABALON	0.35	6.50E-31
BCL2L1	ABALON	0.33	6.61E-28
BIRC6	ABALON	0.55	2.05E-82
EIF2AK2	ABALON	0.31	5.94E-24
GOPC	ABALON	0.31	2.04E-24
KIF5B	ABALON	0.31	6.05E-25
MAPK8	ABALON	0.32	6.24E-26
MTOR	ABALON	0.32	6.32E-27
NAF1	ABALON	0.33	4.50E-28
PIK3C3	ABALON	0.36	1.15E-32
PTEN	ABALON	0.44	2.77E-50
RPS6KB1	ABALON	0.33	2.34E-28
WDFY3	ABALON	0.53	1.88E-76
IL24	AL161431.1	0.34	6.86E-29

*p* < 0.05 was regarded as a significant difference.

**FIGURE 4:**
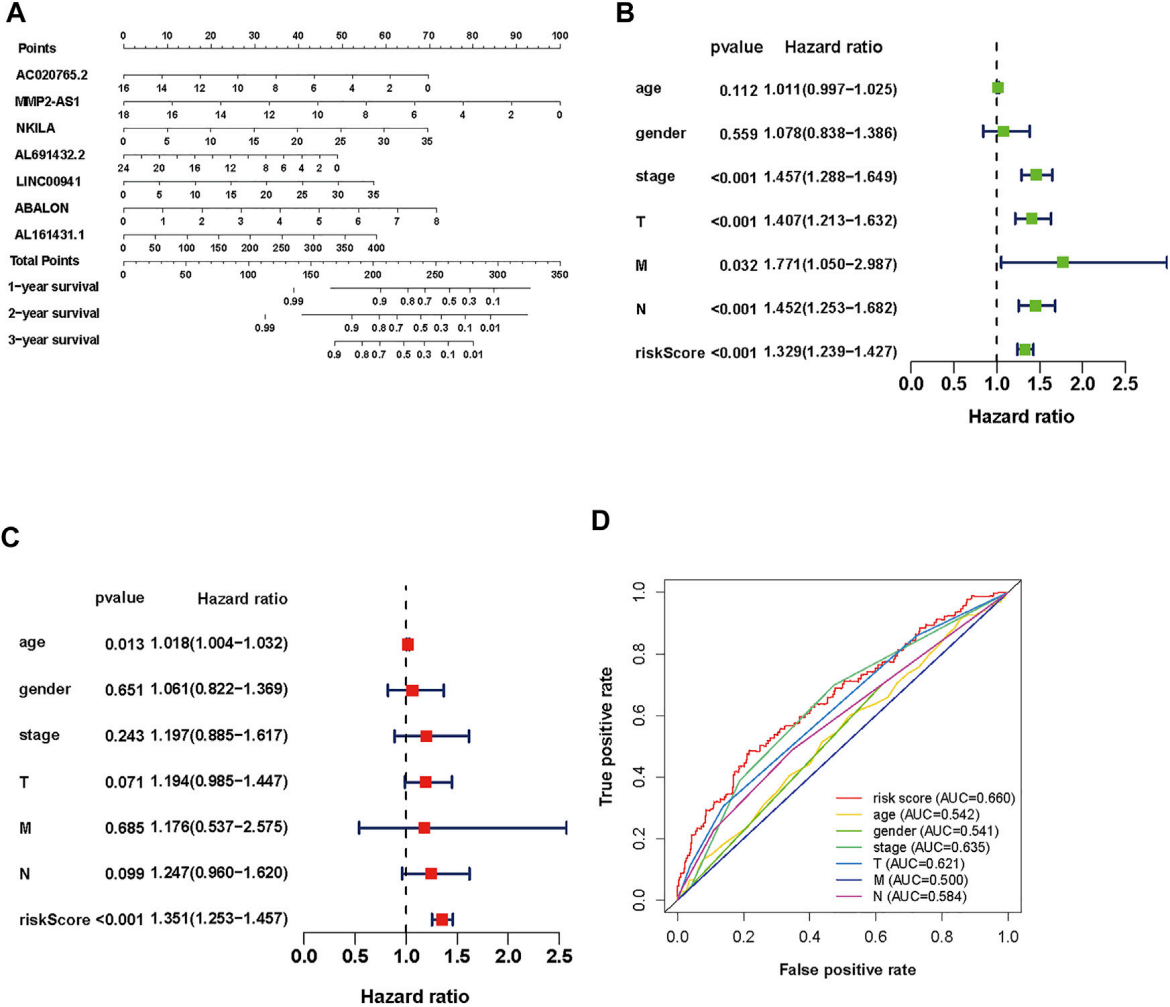
Evaluation of the prognostic risk model of the 7 autophagy-associated lncRNAs in NSCLC. **(A)** Nomogram of the 7 autophagy-associated lncRNAs. **(B,C)** The univariate **(B)** and multivariate **(C)** Cox analysis of risk score model and clinical features. **(D)** The ROC curve analysis displays the prognostic accuracy of clinical features such as age, gender, T stage, M stage, N stage, and risk score.

To establish an accurate prognosis for NSCLC patients, we evaluated the survival rate about 7 autophagy-associated lncRNAs at 1, 2, and 3 years *via* calculating a nomogram. And the nomogram may help specialists make individualized clinical therapy for NSCLC patients (Figure 4A).

### Clinical value of the prognostic autophagy-associated long non-coding RNAs signature

To assess its clinical value, we calculated risk scores about the autophagy-associated lncRNAs model and clinical data from TCGA, including age, gender, grade, and TNM stage. Then, we used univariate and multivariate Cox regression analyses (Figures 4B,C, respectively). The univariate analysis displayed that stage, T stage, N stage, M stage, and the prognostic risk score were closely associated with OS. Then the multivariate analysis revealed that age and risk score were closely associated to OS. We performed multiple ROC curve to assess the accuracy of prediction about the signature. The results displayed that the AUC value of the prognostic risk-related model was 0.660, which was higher than other clinic factors (Figure 4D). In general, the ROC curves indicated that the predictive accuracy of the prognostic lncRNAs model in NSCLC was acceptable.

**FIGURE 5:**
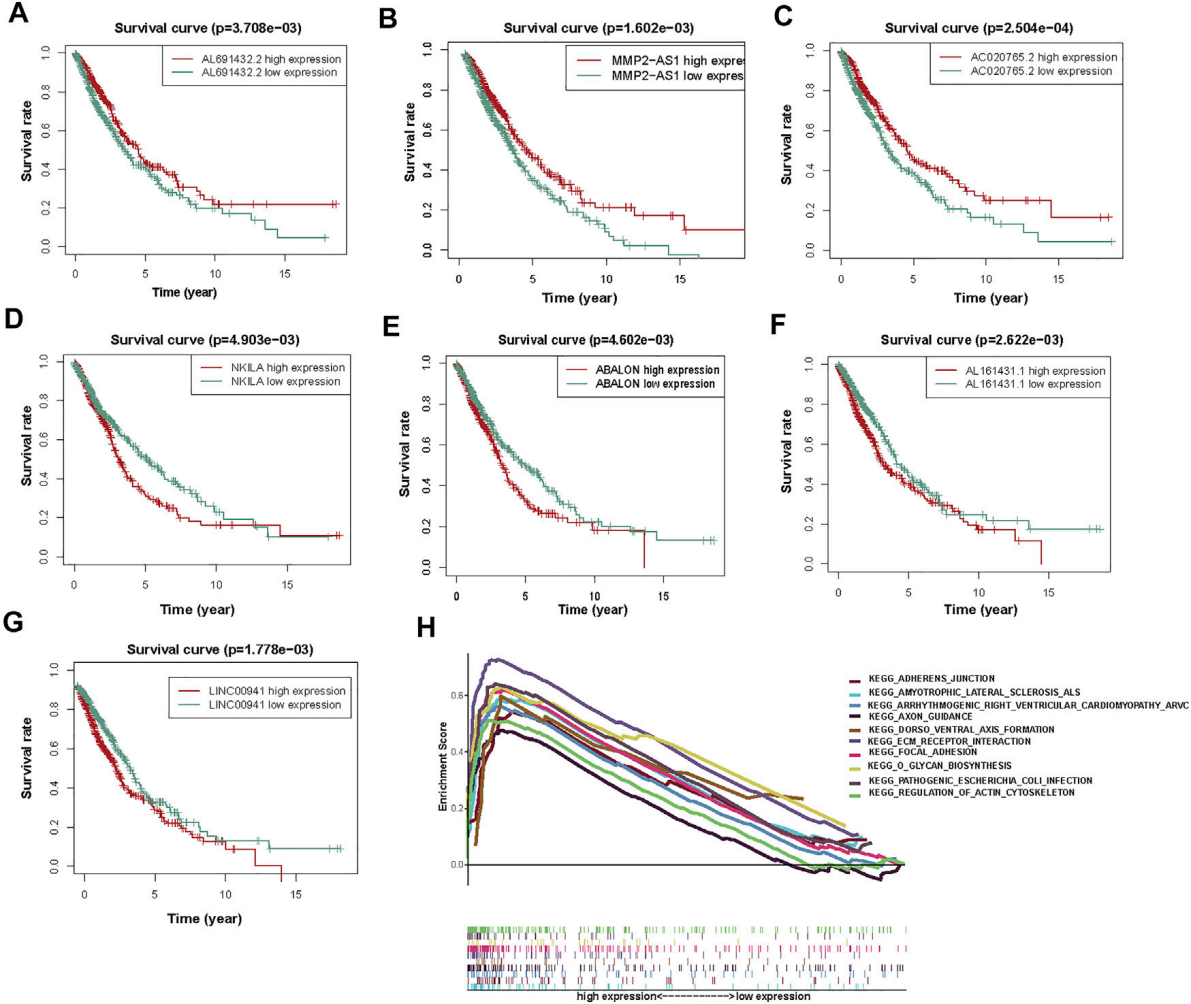
The KM survival curves and GSEA analysis of 7 prognostic autophagy-associated lncRNAs. **(A)** AL691432.2, **(B)** MMP2-AS1, and **(C)** AC020765.2 were favorable prognostic factors. **(D)** NKILA, **(E)** ABALON, **(F)** AL161431.1, and **(G)** LINC00941 were harmful prognostic factors. **(H)** Top ten primarily enriched categories of 7 autophagy-associated lncRNAs in the high-risk group by GSEA.

Then, to determine the correlation between autophagy-associated lncRNAs and OS, we drew Kaplan Meier curves. And we identified the seven lncRNAs (AC020765.2, MMP2-AS1, NKILA, LINC00941, ABALON, AL691432.2, and AL161431.1). These results determined the seven autophagy-associated lncRNAs were closely related to the OS of NSCLC patients (Figures 5A–G).

### GSEA enrichment

According to the prognostic model of autophagy-associated lncRNA, GSEA divided the gene set into high-risk and low-risk groups (FDR q-value < 0.05). The result displayed that the high-risk group was primarily enriched in stromal pathways, such as ECM receptor interaction and regulation of actin cytoskeleton. Furthermore, the O-glycan biosynthesis pathway was also important. It is worth noting that the adherens junction pathway was closely associated with the autophagy-associated lncRNAs. These results further confirmed that these autophagy-associated lncRNAs could regulate NSCLC and autophagy by some special pathways, which may inspire new approaches of therapy in NSCLC (Figure 5H).

### Downregulation of apoptotic BCL2L1-antisense long non-coding RNA inhibited proliferation and metastasis in non-small cell lung cancer cells

**FIGURE 6:**
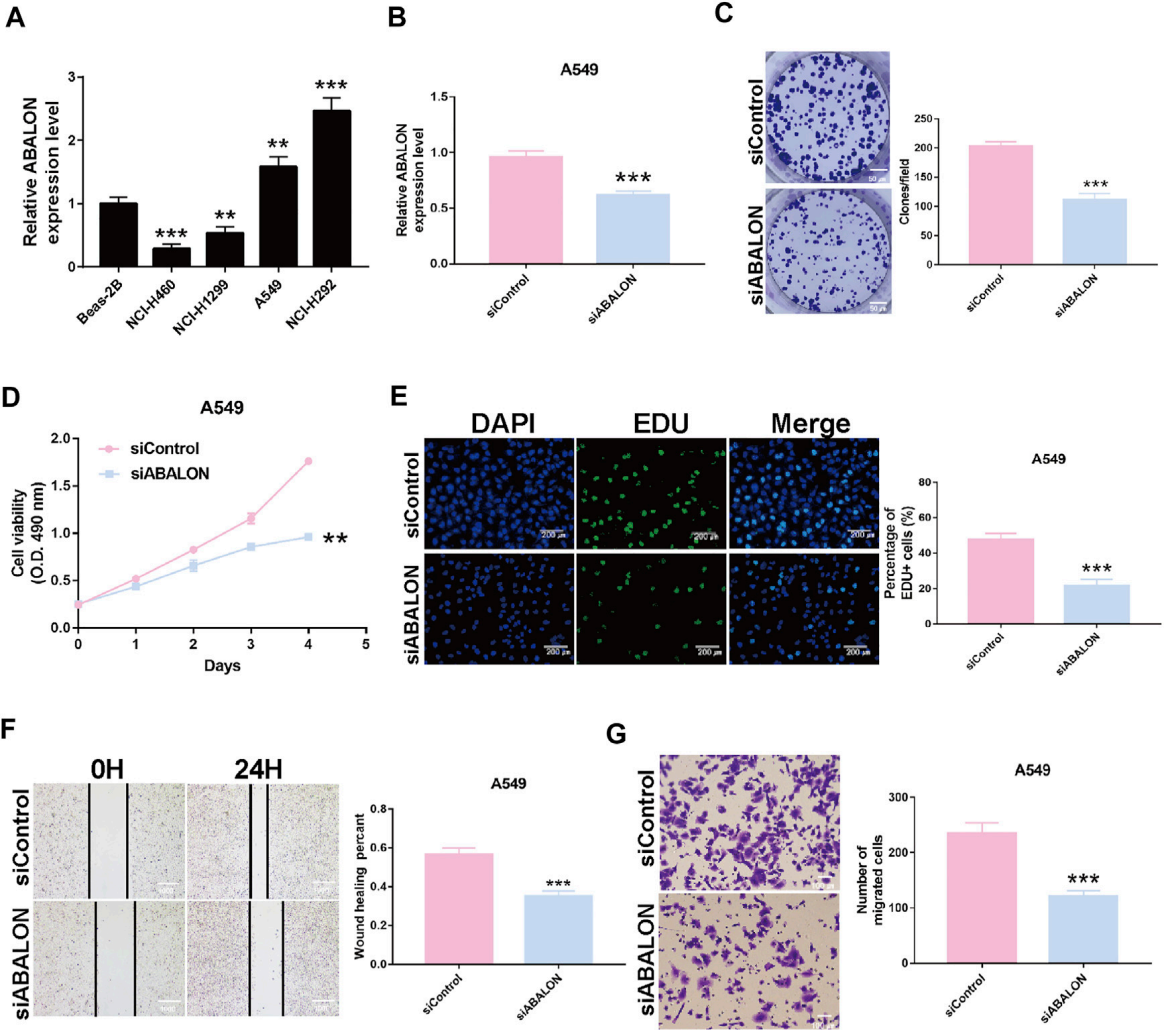
Downregulation of ABALON suppressed the proliferation and metastasis in A549 cells. **(A)** The expression of ABALON in corresponding cell lines identified by RT-qPCR. **(B)** RT-qPCR for the expression of ABALON in siControl and siABALON A549 cells. **(C–E)** Colony formation **(C)**, MTT **(D)**, and EDU **(E)** assays identified proliferation was suppressed. **(F,G)** Cell metastasis suppression was identified by wound healing **(F)**, and transwell **(G)** assays. **p < 0.01, ***p < 0.001.

Survival analysis displayed the ABALON was a risk factor with a poor prognosis. We explored the biological signaling pathways by GSEA, and the adherens junction pathway showed a crucial enrichment score (NES = 2.08, FDR q-val = 0.020). Consequently, we select ABALON for experimental validation. Based on the result of qRT-PCR, we chose two representative NSCLC cell lines (NCI-H292 and A549) to carry out experiments *in vitro* (Figure 6A).

**FIGURE 7:**
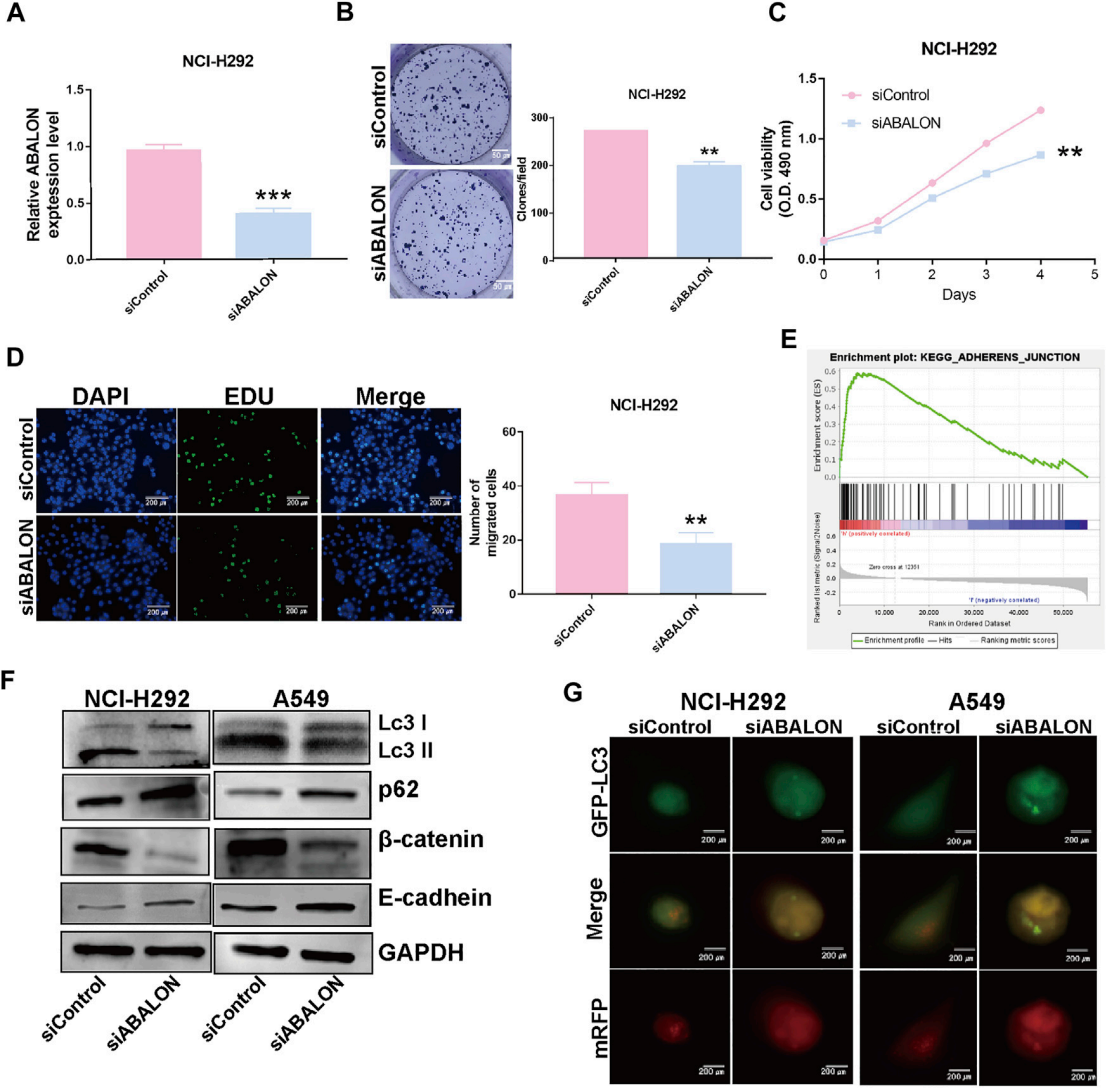
Downregulation of ABALON suppressed the proliferation and metastasis in NCI-H292 cells and promoted autophagy level. **(A)** RT-qPCR for the expression of ABALON in siControl and siABALON NCI-H292 cells. **(B–D)** Colony formation **(B)**, MTT **(C)**, EDU **(D)** assays revealed cell proliferation promotion *via* downregulation of ABALON in NCI-H292 cells. **(E)** A significant enrichment results between ABALON-high and ABALON-low groups in NSCLC. **(F)** Western blotting to illustrate the expression levels of LC3-Ⅱ, P62, E-cadherin and β-catenin. **(G)** Tandem mRFP-GFP fluorescence microscopy to illustrate the levels of autophagy. **p < 0.001.

First, the siRNA of ABALON and Control were transfected into NCI-H292 and A549 cells. The results of MTT, colony formation, and EDU assays showed low-expression of ABALON inhibited the proliferation of NCI-H292 and A549 cells (Figures 6C–E, 7B–D). The results of transwell and wound healing assays further displayed low expression of ABALON inhibited the metastasis of A549 cells (Figures 6F,G). GSEA analysis indicated that ABALON may influence the behavior of NSCLC *via* the adherens junction pathway (Figure 7E). Therefore, the results of western blotting displayed knockdown of ABALON led to decrease expression of ß-catenin and increase expression of E-cadherin (Figure 7F). In summary, these results show that depletion of ABALON can inhibit NSCLC progression.

### Downregulation of apoptotic BCL2L1-antisense long non-coding RNA inhibits autophagy in non-small cell lung cancer cells

To assess changes of autophagy after depletion of ABALON, we used western blotting and tandem mRFP-GFP fluorescence microscopy as the most common approaches. Western blotting was performed to evaluate protein levels of autophagy markers, including LC3 and p62. Depletion of ABALON compared with siControl significantly reduced the LC3-II/LC3-I ratio and simultaneously increased the level of the autophagy substrate p62 (*p* < 0.05) (Figure 7F).

However, the changes in the abundance of these proteins as assessed by western blot analysis cannot fully reflect real alteration of the autophagic flux alterations, which are mainly reflected in autophagosome docking and fusion with lysosomes. Hence, we transferred NSCLC cells with the mRFP-GFP-LC3B plasmid to visualize phagosome-lysosome fusion *via* fluorescence microscopy. Under the acidic and/or proteolytic conditions of the lysosomal cavity, GFP signal becomes sensitive, while mRFP becomes more stable. Hence, when autophagosomes fuse with lysosom, the index of PH in cells would down and the GFP signal is quenched. Colocalization of GFP and mRFP fluorescence in cells means autophagosomes have not fused with lysosomes, indicating that autophagy is blocked. Conversely, if only mRFP fluorescence is visible, there is highly active autophagy. NCI-H292 and A549 cells transfected siABALON only exhibited yellow fluorescence, which confirmed the results of western blotting (Figures 7F,G). These results indicated that siABALON restrained autophagy in NSCLC cells *in vitro*.

## Discussion

Autophagy is the process in which damaged proteins and organelles are engulfed and transported to lysosomes for degradation and circulation. The effect of autophagy seems to be extremely complicated and is difficult to summarize in cancer (Mizushima, 2007; Morel et al., 2017). It can play a tumor suppressor as well as an oncogenic effect. Through regulating functions of proteins and organelles, autophagy can keep the genome stable, reduce normal cell death and prevent tumorigenesis. For example, autophagy can remove senescent organelles and defective proteins (Wang and Klionsky, 2011; Wu et al., 2012). However, autophagy also maintains cancer cell metabolism, promotes tumor genesis and development, and resistance to therapeutic drugs (White, 2012; Poillet-Perez and White, 2019). Hence, we consider that autophagy can improve the therapeutic effects of tumor-targeting drugs and reduce drug resistance.

In this study, we determined 27 autophagy-associated mRNAs in NSCLC, which were associated with the 7 autophagy-associated lncRNAs. Among them, only ATG12, MTOR, ATG7, and PTEN are known to be related to autophagy in NSCLC. Autophagy-related (ATG) proteins play crucial roles in cancer. The numerous ATG proteins and their core complexes including the ULK/Atg1 kinase core complex, ATG9A/Atg9 trafficking system, ATG12/Atg12-conjugation system and LC3/Atg8-conjugation system, exert multiple activities in the autophagy pathway and take part in all processes of autophagy (Li et al., 2020). Among the 4 autophagy-associated genes identified in this study, ATG7, which acts as an essential protein to promote autophagy, was informed to attenuate the proliferation, invasion, and metastasis of NSCLC (Zheng et al., 2018; Cao et al., 2020). He et al. (2020) identified ATG12 was a target of miR-372-3p. ATG12 can promote autophagy and radiosensitivity in lung adenocarcinoma by suppressing miR-372-3p. Furthermore, we found that PTEN and mTOR exhibited a high correlation coefficient with autophagy. PTEN is an important tumor suppressor and the main antagonist of PI3K, which can promote the degradation of AKT (Lim et al., 2015; Jamaspishvili et al., 2018). In addition, miR-181 was found to mediate cisplatin-resistance and attenuate autophagy through the PTEN/PI3K/AKT pathway in NSCLC (Liu et al., 2018). However, the roles of other autophagy-associated mRNAs in NSCLC remain unclear.

Additionally, we identified 7 autophagy-associated lncRNAs in NSCLC patients from TCGA. Notably, lncRNAs have become a key regulatory factor in various cellular processes. Studies show that lncRNAs can also regulate autophagy in cancer. The specific mechanisms through which lncRNAs regulate autophagy can be segmented into three classes: 1) LncRNAs familiarly modulate autophagy through regulating the expression of ATG proteins. 2) LncRNAs regulate tumor genesis through the AKT/mTOR signaling pathway. mTOR forms two different signal complexes, mTOR complex 1(mTORC1) and mTORC2, by binding to a variety of companion proteins. mTORC1 inhibits the initiation of autophagy by phosphorylating ATG13 and autophagy activating kinase (ULK) (Kim and Guan, 2015). 3) LncRNAs can also act as competing endogenous RNAs to modulate miRNAs. Many studies investigated the effect of autophagy and lncRNAs in other cancers, but less research has been done in NSCLC.

Further bioinformatics analysis confirmed that these 7 autophagy-associated lncRNAs were associated with the OS of NSCLC patients. Among them, NKILA, LINC00941, ABALON, and AL161431.1 were risk factors for prognosis of NSCLC, and other lncRNAs (AC020765.2, MMP2-AS1, and AL691432.2) had the opposite effect. Previous studies have shown that LINC00941, also called MSC upregulated factor (lncRNA-MUF), was negatively associated with OS and phosphorylation of the PI3K/AKT signaling pathway in lung adenocarcinoma patients (Wang et al., 2019b). Another study also found that depletion of LINC00941 inhibited EMT and activated Wnt/ß-catenin signaling in hepatocellular carcinoma (Yan et al., 2017). Additionally, Ren et al. informed that LINC00941 promoted the progression of NSCLC through the miR-877-3p/VEGFA axis (Ren et al., 2021). AL161431.1 facilitated the proliferation and metastasis by regulating miR-1252-5p in endometrial carcinoma (Gu and Liu, 2020). Similarly, Qiang et al. found that AL161431.1 was also negatively associated with the OS of patients in lung squamous cell carcinoma (Ju et al., 2020). Matrix metalloproteinases (MMPs) are a family of zinc-dependent endopeptidases, and some studies indicated MMP2 promotes invasion and metastasis of NSCLC cells (Hsieh et al., 2019). Thus, we speculate that MMP2-AS1, encoding MMP2 antisense RNA1, may restrain the function of MMP2 in NSCLC. Consistent with our finding, LINC00941 tended to be a high-risk factor and AL691432.2 tended to be a low-risk factor in the construction of a prognostic model for NSCLC.

E-cadherin is an important cyctomembrane component and plays an important role in adherhens junction. E-cadherin and the Catenins (β-catenin, α-catenin, p120-catenin) are the substates of important kinases and phosphatases for regulating adherens junction (Coopman and Djiane, 2016). In this study, we demonstrated that apoptotic BCL2L1-antisense LncRNA (ABALON) acts as an oncogene in NSCLC *via* adherens junction pathway. We also confirmed that ABALON promoted autophagy by western blotting and fluorescence microscopy. However, the mechanisms of ABALON need further study. In summary, the results reveal the 7 identified autophagy-associated lncRNAs have a prognostic value in NSCLC.

However, our study has certain limitations. First, we applied traditional statistical analysis methods to establish and evaluate prognostic risk models for 7 autophagy-associated lncRNAs. Although these methods have been applied and validated in many studies, we need to refine our further studies with more advanced methods and techniques in the future. In the study, we confirmed ABALON promotes the proliferation, metastasis and autophagy in NSCLC cells *via* biological experiments. However, the mechanism by which ABALON regulates NSCLC cells remains unclear. To further verify our prediction results, in-depth studies about the molecular mechanisms are needed.

## Conclusion

In conclusion, this study identified 26 autophagy-associated genes in NSCLC, and constructed a prognostic risk signature of 7 autophagy-associated lncRNAs. The signature was accurate to predict prognosis of NSCLC patients with high reliability. Moreover, experimental validation confirmed that ABALON promotes the proliferation, metastasis, and autophagy in NSCLC cells. This signature provides a basis for further studies on the clinical application of these autophagy-associated lncRNAs. In the future, with prospective validation, the 7 autophagy-associated lncRNAs signature may improve predictive accuracy and guide individualized therapy for non-small cell lung cancer patients.

## Data Availability

The original contributions presented in the study are included in the article/Supplementary Material, further inquiries can be directed to the corresponding authors.
