# Supplementary material for: An autophagy-associated lncRNAs model for predicting the survival in non-small cell lung cancer patients
Source: Front Genet. 2022 Sep 2;13:919857. doi: 10.3389/fgene.2022.919857 (PMC9479339; doi:10.3389/fgene.2022.919857)
Supplement: Supplementary file 1 [file Presentation1.pdf]

Antibodies, reagents, plasmids and small interfering RNA

The ABALON gene-specific short interfering (siRNA), and non-specific control siRNA (siControl) were purchased from RiboBio (Shanghai, China). Antibodies against, GAPDH, LC3, p62 (Cell Signaling Technology, Beverly, MA, USA) and β-catenin, E-cadherin (Abcam, Cambridge, MA, USA) were used.

Proliferation Assays

For EDU assay, firstly siABALON and siControl were transfected into NCI-H292 and A549 cell. After 48 h, cells were incubated with 25 μM EDU for 2 h. Discard the supernatant, wash with phosphate buffered saline (PBS) for 2-3 times, then the cells were fixed with 4% formaldehyde for 30 min at room temperature and decolorized with 0.5% Triton X-100 for 10 min. After washing with PBS, the cells were dyed with Apollo reaction cocktail for 30 minutes, Discarding the supernatant, the cells were washed with 0.5% Triton X-100-PBS for 2-3 times. Then the cells were stained with Hoechst 33342 for 30 min and observed under fluorescence microscope.

For MTT assay, siABALON and siControl were transfected into NCI-H292 and A549 cell. After 24 h, cells were seeded into 96-well plates. Add MTT to 96 well plate, 20μl per well, and put it into incubator for further culture for 4 h. Then the 96-well plate was centrifuged for 5min at 4,000 rpm. The medium was removed, and add DMSO to 96 well plate, 150μl per well. The absorbance at 570 nm was detected.

For plate colony formation assay, siABALON and siControl were transfected into NCI-H292 and A549 cell. After 24 h, cells were seeded in 6-well. After about 3 weeks, discard the medium, wash with PBS for 2-3 times. Then the colonies were fixed with 10% formalin for 15 min and washed with PBS, followed by dyeing with hematoxylin. The number of colonies was counted.

Migration assay

For Transwel assay, A total of 50,000 cells were seeded to the upper chamber, and medium containing 20% FBS was added into the lower chamber. 16 hours later, the chamber were fixed with 20% methanol for 20 min and stained with crystal violet for 20 min. The chamber was washed with PBS. Then these membranes were carved. The number of migrating cells was counted under a microscope.

For wound healing assay, 24h after transfection, the cells were seeded in six-well plates and cultured until cells density up to 90%. Wounds were made in the cell monolayer by a 20μL pipette tip and plates were put into incubator for further culture. After 10 h, 16 h and 24 h, Plates were washed with fresh medium and photographed under a microscope.
